# Serial assessment of biochemical parameters of red cell preparations to evaluate safety for neonatal transfusions

**Published:** 2010-12

**Authors:** Somnath Mukherjee, Neelam Marwaha, Rajendra Prasad, Ratti Ram Sharma, Beenu Thakral

**Affiliations:** *Department of Transfusion Medicine, Postgraduate Institute of Medical Education & Research, Chandigarh, India*; * *Department of Biochemistry, Postgraduate Institute of Medical Education & Research, Chandigarh, India*

**Keywords:** Biochemical changes, packed red cells, whole blood

## Abstract

**Background & Objectives:**

Neonatologists often prefer fresh blood (<7 days) for neonatal transfusions. The main concerns for stored RBCs are ex vivo storage lesions that undermine red cell functions and may affect metabolic status of neonatal recipients. This study was designed to evaluate serial *in vitro* changes of biochemical parameters in different RBC preparations during storage to consider for neonatal transfusions even after storage beyond one week.

**Methods:**

Twenty five units each of whole blood (CPDA-1 RBC, SAGM RBC) were selected for serial biochemical parameter assessment after each fulfilled the quality criteria (volume and haematocrit). These units were tested serially for supernatant potassium, *p*H, lactate, haemoglobin, glucose and red cell 2,3 diphosphoglycerate (2,3 DPG) up to 21 days of storage.

**Results:**

Within each group of RBC, rise in mean concentration of potassium, lactate and plasma haemoglobin from day 1 to 21 of storage was significant in CPDA-1 RBC having the highest levels at day 21. From day 3 to 21, SAGM RBC had higher mean *p*H value than CPDA-1 RBC though this difference was not statistically significant. SAGM RBC had highest mean glucose concentration during storage than other two types of red cell preparations (*P*<0.005). Within each group, fall in mean 2,3 DPG concentration from day 1 to 7 was significant (*P*<0.05). A positive correlation existed between mean plasma potassium and haemoglobin in all three types of red cells (r=0.726, 0.419, 0.605 for CPDA-1 RBC, SAGM RBC and whole blood respectively, *P*<0.005).

**Interpretation & Conclusions:**

All the three red cell preparations tested revealed biochemical changes within acceptable limits of safety till 21 days of storage. CPDA-1 RBCs had the highest degree of these changes.

Red blood cells (RBC) are ideally suited to their primary function *i.e*., transport of oxygen from lungs to the tissues and carbon dioxide from the tissues to lungs. In neonates, RBC transfusion therapy is required in various clinical situations. During the first week of life, neonates experience a decline of RBCs caused by both physiologic factors and in sick premature infants due to sepsis, necrotizing enterocolitis or phlebotomy blood losses. Apart from this, neonates may also need to undergo surgical procedures. In most of these situations neonates usually require repeated small volume transfusions (10-15 ml/kg body weight)^1^ with RBCs suspended either in citrate phosphate dextrose adenine-1 (CPDA-1) solution at a haematocrit of approximately 70 per cent or in extended storage media (additive solution) at a haematocrit of approximately 60 per cent. Large volume transfusion (>25 ml/kg) are required in specific situations, e.g., exchange transfusion in hyperbilirubinaemia; exchange transfusion for sepsis, extracorporeal membrane oxygenation (ECMO) and cardiac bypass surgery for congenital heart disease^2^,^3^.

Many controversies exist regarding neonatal transfusion practice. Neonatologists often insist on transfusion of fresh RBC (<7 days old) because of various concerns regarding stored RBCs. There is an increase in extracellular potassium (K^+^), decrease in *p*H and 2,3 diphosphoglycerate (2,3 DPG) in stored blood which is important for oxygen release in the tissues. There is also a possible risk of use of compounds like mannitol and glucose, in relatively large amounts, which are present in RBC additive solutions. However, when neonates require repeated transfusions, requisitions for fresh blood may lead to multiple allogeneic blood donor exposure and its consequent risks. Certain *in vitro* studies have revealed that alterations in various biochemical parameters on storage of whole blood/RBC do not significantly affect neonatal homeostasis after small volume or top-up transfusions^4^,^5^. These observations may help in reducing multiple donor exposures in neonates who require repeated small volume transfusions. One donor unit could be dedicated for one neonate after aliquotting it into small volumes^6^.

However, before changing neonatal transfusion practices in our scenario we need to study biochemical changes in various red cells preparation on storage. Hence this study was designed to evaluate the serial *in vitro* changes of the biochemical parameters in different RBC preparations during storage before considering their use for neonatal transfusion even after storage beyond one week.

## Material & Methods

The study was conducted by the Department of Transfusion Medicine Postgraduate Institute of Medical Education & Research (PGIMER), Chandigarh, a tertiary care hospital, in collaboration with the Department of Biochemistry during the period from January 2007 to December 2007. Blood donors were screened as per regulations of Drugs and Cosmetics Rules, Govt. of India^7^. Phlebotomy was performed after taking consent from donor. The study protocol was cleared by the Ethics committee of the Institute.

Twenty five units whole blood were collected in single plastic blood bags (J. Mitra Industries Pvt. Ltd. Haryana, India), 25 units of RBC suspended in citrate, phosphate, dextrose, adenine (CPDA-1) prepared in double blood bags (J. Mitra Industries Pvt. Ltd. Haryana, India) and 25 units of RBC suspended in additive solution composed of saline, adenine, glucose and mannitol (SAGM) were prepared in quadruple (Top and Top) bags (Teumo Penpol Ltd. Trivandrum, India) as per standard procedures^8^.

Quality check as per Directorate General Health Services criteria (DGHS)^9^. The initial quality of RBC preparations was assessed by utilizing the quality control parameters (visual examination of bag, volume and haematocrit) laid down by DGHS (Table I).

**Table I:** Results of quality control parameters in different red cell preparations

Parameters				
	DGHS criteria*		Present study	
	Volume (ml)	Haematocrit (%)	Volume (ml) (mean ± SD)	Haematocrit (%) (mean ± SD)
CPDA-1 RBC	280 ± 40	70 ± 5	281.8 ± 21.1	69.6 ± 3.9
SAGM RBC	350 ± 20	60 ± 5	347 ± 12.2	60.4 ± 2.9
Whole blood	350 ± 10%	40 ± 5	351.2 ± 8.3%	42.8 ± 4.1

* Directorate General Health Services, Transfusion Medicine Technical Manual 2003^9^

### 

#### Biochemical parameters:

Twenty five units each of whole blood, CPDA-1 RBC, SAGM RBC were tested serially for the following biochemical parameters: supernatant potassium (K^+^), *p*H, lactate, haemoglobin, glucose and red cell 2,3 DPG up to 21 days of storage. Supernatant plasma potassium and plasma glucose level were estimated by automated analyzer (Hitachi 902, Roche, Germany). Supernatant plasma lactate was estimated by commercial kits (Randox Laboratories Limited, Ardmore, Diamond Road, UK), with the help of colorimeter. Plasma haemoglobin was determined by Hemcue AB (SE-26223, Angelholm, Sweden). Red cell 2,3 DPG was estimated by commercial kits (Roche Diagnostics, 38289, Manheim, Germany), with the help of UV spectrophotometer. Ten samples of cord blood red cells were also subjected to 2, 3, DPG estimation. *p*H was measured by using digital *p*H meter (spectrophotometer, Indian Laboratory and Scientific Instrument Ltd., Chandigarh).

Plasma potassium (K^+^) and *p*H were estimated daily from day 1 till day 21. Estimation of glucose, lactate and plasma haemoglobin was performed at weekly intervals till three weeks. 2,3 DPG was estimated on day 1, 7, and 14 of storage.

#### Statistical analysis:

ANOVA was applied for comparison of biochemical changes in the RBCs among intra- and inter-group blood bags. Independent T test was applied when significant difference was found. To assess the correlation between biochemical parameters of different red cell preparations during storage, Pearsons correlations coefficient (r) was applied. *P*<0.05 was considered statistically significant. All analyses were performed with the software package SPSS Version 13.0 for windows (USA).

## Results

The quality parameters such as blood volume and haematocrit of all the units included in the study conformed to the standards established by DGHS^9^. There was a significant rise in K^+^concentration (*P*<0.001) from day 1 to day 21 of storage. CPDA-1 RBC had the highest significant mean plasma K^+^during storage (*P*<0.05). SAGM RBC had slightly higher mean plasma K^+^than whole blood from day 7 till 21. Mean *p*H fall was observed in each group of RBC and the *p*H change was also significant (*P*<0.001) during storage for each group of RBC. On day 1 CPDA-1 RBC had significantly lower *p*H than both whole blood and SAGM RBC (*P*<0.05). From day 3 to day 21, SAGM RBC had slightly higher mean *p*H value than CPDA-1 RBC though this difference was not statistically significant (Table II). Whole blood had significantly higher mean *p*H (*P*<0.005) than both SAGM RBC and CPDA-1 RBC. Fall in mean glucose concentration was noticed in each group RBC from day 1 to day 21 of storage and it was significant (*P*<0.005). SAGM RBC had significantly higher mean glucose concentration during storage than other two types of RBC preparations (*P*<0.005). Whole blood had higher mean glucose concentration than CPDA-1 RBC, but the difference was significant only from day 7 to 21 of storage. There was also significant rise (*P*<0.005) in mean lactate concentration during storage for each group of red cells. Mean lactate value was significantly higher in CPDA-1 RBC on day 7, 14 and 21 than the other red cells (*P*<0.05). However, mean lactate concentration was not significantly different between whole blood and SAGM RBC. Within each group of RBC fall in mean 2,3 DPG concentration from day 1 to 7 was significant (*P*<0.001). 2,3 DPG concentration was less than 1µmol/g Hb in CPDA-1 RBC and SAGM RBC on day 14 of storage. Ten samples of cord blood were taken as negative control to estimate red cell 2,3 DPG values. 2,3 DPG was undetectable in all the cord blood samples tested.

**Table II:** Results of serially assessed biochemical parameters (mean ± SD) in each red cell preparations

Parameter	CPDA-1 RBC (n=25)				SAGM RBC (n=25)				Whole blood (n=25)			
Days of storage	1	7	14	21	1	7	14	21	1	7	14	21
Mean *p*H (room temp.)	7.10 ± 0.1	6.7 ± 0.1	6.5 ± 0.1	6.3 ± 0.1*	7.2 ± 0.1	6.8 ± 0.1	6.6 ± 0.1	6.4 ± 0.1*	7.3 ± 0.1	6.9 ± 0.1	6.7 ± 0.1	6.5 ± 0.1*
Mean plasma K+ (mEq/l)	6.4 ± 1	15.8 ± 2.7	24.3 ± 5.9	37.8 ± 5.7*	4.01 ± 0.8	9.1 ± 1.6	17.3 ± 3.5	25.2 ± 4.5*	4.23 ± 0.7	9.2 ± 1.7	15.7 ± 3.3	22.8 ± 5.2*
Mean 2,3 DPG µmol/g Hb (% of initial)	11.5 ± 6.2 (100%)	4.1 ± 1.6 (36%)*	0.4 ± 0.2 (3.5%)**	N D	10.2 ± 3.9 (100%)	4.5 ± 0.2 (44%)*	0.7 ± 0.2 (6.8%)**	ND	14.1* ± 4.4 (100%)	6.1 ± 2.3 (43.4%)*	2.2 ± 0.9 (15.5%)**	ND
Mean plasma lactate+ (mg/dl)	51.6 ± 16.7	106.4 ± 17.1	127.4 ± 14.1	141.9 ± 16.6	47.3 ± 10.9	95.6 ± 17.3	109.3 ± 21.9	128.5 ± 10.8	44.1 ± 14.2	88.4 ± 12.8	114.1 ± 16.1	130.9 ± 14.6
Mean plasma glucose (mg/dl)#	350.3 ± 62.6	252.1 ± 46.4	173.4 ± 48.1	116.2 ± 33.1	426.3 ± 50	358.4 ± 40.2	310.1 ± 43.5	252.7 ± 40.3	372.1 ± 52.7	292.8 ± 51.1	228.2 ± 59.4	159.7 ± 60.6
Mean plasma haemoglobin (mg/dl)##	39.7 ± 13.1	85.9 ± 20.1	124.4 ± 18	142.6 ± 14.5	34.5 ± 13.2	77.9 ± 22	104.9 ± 26.1	128.8 ± 19.8	28.1 ± 12.6	57.5 ± 24.9	83.8 ± 24.6	113.7 ± 23.3

ND, not done.

* *P* <0.001 compared to day 1

** *P* <0.001 compared to day 7; mean value from day 1 to 7 *P*<0.05, from day 7 to 14 and 14 to 21 *P*<0.001 for all RBC;

# mean value from day 1 to 21 *P* <0.005 for all RBC;

## mean value from day 1 to 21 *P*<0.005.

Within each group of red cells rise in mean plasma haemoglobin concentration from day 1 to 21 was significant (*P*<0.005) (Table II). Mean plasma haemoglobin was significantly less (*P*<0.005) in whole blood as compared to CPDA-1 RBC and SAGM RBC during storage. However, the plasma haemoglobin level rose significantly in CPDA-1 RBC on last day of storage.

There was significant negative correlation between mean *p*H versus mean plasma lactate and plasma glucose versus plasma lactate in each group of red cells during storage. (r= -0.714, -0.732, -0.874, and -0.778, -0.869, -0.696 respectively for CPDA-1 RBC, SAGM RBC and whole blood respectively, *P*<0.005). Significant positive correlation existed between mean plasma K^+^and plasma haemoglobin in all three types of red cells (r=0.726, 0.419, 0.605 for CPDA-1 RBC, SAGM RBC and whole blood respectively, *P*<0.005). Significant positive correlations were observed between mean *p*H and 2, 3 DPG (r=-0.623, 0.761 and 0.766 for CPDA-1 RBC, SAGM RBC and whole blood respectively). However, significant negative correlation were observed between lactate and 2,3 DPG concentration (r= -0.698, -0.716 and -0.764 for CPDA-1 RBC, SAGM RBC and whole blood respectively *P*<0.005) between plasma K^+^and 2,3 DPG (r= -0.694, -0.761, -0.751 respectively for CPDA-1 RBC, SAGM RBC and whole blood.

## Discussion

The main concerns for stored RBCs are ex vivo storage lesions that undermine red cell functions and may affect the metabolic status of the *in vivo* milieu of the neonatal recipients^10^,^11^. Additional possible risks are also from the additives like glucose, mannitol which are present in red cells in large amount^12^. However, when repeated top up transfusions are required, each fresh unit increases the donor exposure for neonate and its subsequent risks of developing transfusion transmitted diseases.

In the present study significant rise in supernatant K^+^was seen in the three RBC preparations on storage similar to other studies^12^,^13^. In a study by Strauss^2^, the supernatant plasma level after 42 days of RBC storage in additive solution rose to 50 meq/litre. However, the actual dose of bioavailable K^+^transfused (ionic K^+^in the volume of extracellular fluid) during small volume transfusion is very low. It has been estimated that the K^+^concentration of CPDA-1 RBC at haematocrit of 70 per cent at 35 days of storage (permitted shelf life) will be around 70-80 meq/litre. The transfusion dose in a neonate is 15 ml/kg and in a one kg neonate only 0.3 to 0.4 meq K^+^will be infused. This dose is even smaller than the usual daily requirement of 2-3 meq/kg. However, this rationale will not apply to large volume transfusions (>25 ml/kg) such as for exchange transfusions. As expected, there was a significant positive correlation between plasma K^+^and haemoglobin levels in all the three RBC preparations and was in agreement with earlier studies^13^,^14^. The plasma haemoglobin values were higher in CPDA-1 RBC as compared to leukoreduced SAGM RBC. Possible explanation for less hemolysis in SAGM RBC was due to presence of membrane stabilizers such as mannitol or citrate in the additive solutions^15^,^16^. The degree of haemolysis was well below 0.8 per cent, the permissible value at the end of shelf life of all RBC preparations.

Glucose is the main source of energy for red cell metabolism via glycolytic pathway. In blood bags the glucose concentration is limited and as glucose is utilized, there is a concomitant ATP (adenosine triphosphate) depletion and decrease in red cell viability. We observed a fall in glucose on storage in all the three RBC preparations, but SAGM RBC had significantly higher glucose concentration than whole blood. The highest glucose concentration in SAGM RBC was due to additional 900 mg dextrose present in 100 ml of additive solution. This helps to prolong the shelf life of RBCs by ATP generation through glycolytic pathway^17^.

Lactate, the end product of anaerobic metabolism of red cells increased during storage. The glucose utilization and lactate production were negatively correlated in all the red cell preparations with CPDA-1 RBC having highest lactate concentration on day 21. Possible explanation may be due to less quantity of adenine and other nutrients present in CPDA-1 RBCs for ATP generation as compared to SAGM RBC or whole blood. *p*H is an important marker of RBC metabolism during storage which slows as *p*H falls. A mathematical deduction of the *p*H curve of many samples of stored blood in various storage solutions revealed a lower limit of *p*H 6.2 below which RBCs had decreased ATP generation^18^. Though ATP measurement was not done in the present study, by day 21, CPDA-1 RBC, SAGM RBC and whole blood mean *p*H was above the lower limit of *p*H threshold of 6.2, thus ATP generation would most likely be persisting in all the red cell preparations.

In the present study a significant decline was found in 2,3 DPG levels in all the red cells by 2 wk of storage with a significant positive correlation with *p*H. Beutler^19^,^20^ also found 2,3 DPG to be totally depleted from RBCs by 21 days of storage. Although the levels decline during storage, they increase rapidly after transfusion in the recipient. This regeneration of 2,3 DPG is also supported by a study which observed that red cells stored for 3 wk were as efficacious as erythrocytes of 3.5 h of storage in reversing neurocognitive deficit of acute anemia^21^. The p50 of transfused adult RBCs increases rapidly as compared to low p50 values of preterm infant’s endogenous red cells due to high foetal Hb concentration. Moreover, cord blood has undetectable 2,3 DPG levels (similar result was observed by us on cord blood sample) which along with high foetal Hb concentration cause difficulty in offloading oxygen to the tissues. Thus, transfusing the stored adult RBCs to preterm infants still has an advantage over endogenously produced infant’s own RBCs.

In conculsion, results from the present study indicate and substantiate the fact that biochemical alterations do occur in stored red cells. The three red cell preparations tested revealed these changes within acceptable limits of safety till 21 days of storage. Though, whole blood had least biochemical alterations followed by SAGM RBC, but for transfusing the same amount of blood (15 ml/kg) for correcting anaemia, CPDA-1 or SAGM RBC are preferred over whole blood because of higher post-transfusion haemoglobin increment by RBCs as compared to whole blood. Additional benefits like pre-storage leucoreduction and better inventory management are possible with RBC preparations and various adverse effects of fresh whole blood, both immunological and cytomegalovirus transmission can be minimized. Though, CPDA-1 RBCs had highest degree of alterations, these changes need to be considered in light of their effect on neonatal top-up transfusions. A pilot *in vivo* study needs to be carried out to confirm the post-transfusion safety in neonates. A double blind multi-center randomized control trial is ongoing out in Canada to study the effectiveness of age of red cells (stored vs. fresh) in neonates requiring at least one transfusion^22^ with the aim to determine if RBCs stored for 7 days or less (fresh RBCs) compared to the practice of using dedicated single units of donated RBCs decrease major nosocomial infections and organ dysfunction in neonates.

## References

[1] Brecher ME (2005). *Neonatal and pediatric transfusion practice. Technical manual*.

[2] Strauss RG (2000). Data-driven blood banking practices for neonatal RBC transfusions. *Transfusion*.

[3] Levy GJ, Strauss RG, Hume H, Schloz L, Albanese MA, Blazina J (1993). National survey of neonatal transfusion practices: I. Red blood cell therapy. *Pediatrics*.

[4] Strauss RG, Sacher RA, Blazina JF, Blanchette VS, Schloz LM, Butch SH (1990). Commentory on small-volume red cell transfusions for neonatal patients. *Transfusion*.

[5] Brecher ME (2000). *Collected questions and answers*.

[6] Strauss RG, Anderson KC, Ness PM (2000). Neonatal transfusion. *Scientific basis of transfusion medicine: Implications for clinical practice*.

[7] Malik V (2003). *Drugs and Cosmetic Act, 1940*.

[8] Brecher ME (2005). *Preparation of red blood cell*, Method 6.4. AABB, Technical manual.

[9] Saran RK (2003). *Transfusion medicine*, Technical Manual.

[10] Strauss RG (2006). Controversies in the management of the anemia of prematurity using single-donor red blood cell transfusions and/or recombinant human erythropoietin. *Transfus Med Rev*.

[11] Hess JR, Greenwalt TG (2002). Storage of red blood cells: new approaches. *Transfus Med Rev*.

[12] Sawant RB, Jathar SK, Rajadhyaksha SB, Kadam PT (2007). Red cell hemolysis during processing and storage. *Asian J Transfu Sci*.

[13] Seghatchian MJ, Vickers M, Ip AH, Stivala JF, de Silva PM (1993). The potassium haemoglobin and acid content of CPDA-1 whole blood, plasma reduced red cells and red cells suspended in SAG-M over 35 days. *Transfus Med*.

[14] Hall TL, Barnes A, Miller JR, Bethencourt DM, Nestor L (1993). Neonatal mortality following transfusion of red cells with high plasma potassium levels. *Transfusion*.

[15] Strauss RG (1991). Transfusion therapy in neonates. *Am J Dis Child*.

[16] Simon ER (1962). Red cell preservation: further studies with adenine. *Blood*.

[17] Nakao K, Wada T, Kamiyama T, Nakao M, Nagano K (1962). A direct relationship between adenosine triphosphate-level and *in vivo* viability of erythrocytes. *Nature*.

[18] Valeri CR, Hirsch NM (1969). Restoration *in vivo* of erythrocyte adenosine triphosphate, 2,3-diphosphoglycerate, potassium ion, and sodium ion concentrations following the transfusion of acid-citrate-dextrose-stored human red blood cells. *J Lab Clin Med*.

[19] Beutler E, Anderson KC, Ness PM (1994). Scientific basis of transfusion medicine: Implications for clinical practice. *Scientific basis of transfusion medicine: Implications for clinical practice*.

[20] Beutler E, Petz LD, Swisher SN (1989). Erythrocyte metabolism and its relation to the liquid preservation of blood. *Clinical practice of transfusion medicine*.

[21] Weiskopf RB, Feiner J, Hopf H, Lieberman J, Finlay HE, Quah C (2006). Fresh blood and aged stored blood are equally efficacious in immediately reversing anemia-induced brain oxygenation deficits in humans. *Anesthesiology*.

[22] Fergusson D, Hutton B, Hogan DL, LeBel L, Blajchman MA, Ford JC (2009). The age of red blood cells in premature infants (ARIPI) randomized controlled trial: study design. *Transfus Med Rev*.

